# Assessment of the Oxidative Damage and Genotoxicity of Titanium Dioxide Nanoparticles and Exploring the Protective Role of Holy Basil Oil Nanoemulsions in Rats

**DOI:** 10.1007/s12011-022-03228-0

**Published:** 2022-04-13

**Authors:** Mohamed F. Sallam, Helmy M. S. Ahmed, Aziza A. El-Nekeety, Kawthar A. Diab, Sekena H. Abdel-Aziem, Hafiza A. Sharaf, Mosaad A. Abdel-Wahhab

**Affiliations:** 1 Pharmacology and Toxicology Department, Faculty of Pharmacy, Ahram Canadian University, Giza, Egypt; 2 Toxicology & Pharmacology Department, Faculty of Pharmacy, Cairo University, Cairo, Egypt; 3 Food Toxicology & Contaminants Department, National Research Center, Dokki, Cairo, Egypt; 4 Genetics and Cytology Department, National Research Center, Dokki, Cairo, Egypt; 5 Cell Biology Department, National Research Center, Dokki, Cairo, Egypt; 6 Pathology Department, National Research Center, Dokki, Cairo, Egypt

**Keywords:** Titanium dioxide nanoparticles, Oxidative damage, Genotoxicity, Basil essential oil, Nanoemulsions, Antioxidant

## Abstract

This study was designed to evaluate the oxidative damage, genotoxicity, and DNA damage in the liver of rats treated with titanium nanoparticles (TiO_2_-NPs) with an average size of 28.0 nm and *ξ*-potential of − 33.97 mV, and to estimate the protective role of holy basil essential oil nanoemulsion (HBEON). Six groups of Male Sprague–Dawley rats were treated orally for 3 weeks as follows: the control group, HBEO or HBEON-treated groups (5 mg/kg b.w), TiO_2_-NPs-treated group (50 mg/kg b.w), and the groups treated with TiO_2_-NPs plus HBEO or HBEON. Samples of blood and tissues were collected for different analyses. The results revealed that 55 compounds were identified in HBEO, and linalool and methyl chavicol were the major compounds (53.9%, 12.63%, respectively). HBEON were semi-round with the average size and *ζ*-potential of 120 ± 4.5 nm and − 28 ± 1.3 mV, respectively. TiO_2_-NP administration increased the serum biochemical indices, oxidative stress markers, serum cytokines, DNA fragmentation, and DNA breakages; decreased the antioxidant enzymes; and induced histological alterations in the liver. Co-administration of TiO_2_-NPs plus HBEO or HBEON improved all the tested parameters and the liver histology, and HBEON was more effective than HBEO. Therefore, HEBON is a promising candidate able to protect against oxidative damage, disturbances in biochemical markers, gene expression, DNA damage, and histological changes resulting from exposure to TiO_2_-NPs and may be applicable in the food and pharmaceutical sectors.

## Introduction

Nanotechnology is the most important technology in the twenty-first century which has made tremendous breakthroughs in nanomaterials development for the advancement of biotechnology and medical research fields [1]. Titanium dioxide NPs (TiO_2_-NPs) are widely utilized in different applications owing to their high stability, anticorrosion, and photocatalytic characteristics [2, 3] such as nutraceuticals, pharmaceuticals, toothpaste, cosmetics, paper industries, paints production, and food products.

Several reports postulated that TiO_2_-NPs induce oxidative stress, cell apoptosis, DNA damage, and inflammatory reactions [4, 5]. The toxicity of TiO_2_-NPs is depending on the exposure conditions, the particle sizes, and the zeta potential [6]. Exposure to TiO_2_-NPs occurs by inhalation, ingestion, injections, or skin contact then accumulates in several organs and tissues by circulating blood [7, 8]. The International Agency for Research on Cancer (IARC) classified TiO_2_-NPs as group 2B (probably carcinogenic to humans) [5]. Recent studies indicated that TiO_2_-NPs induce severe toxicities to different organs such as the liver and the kidney after the absorption by the GIT [3, 9]. Several in vivo and in vitro researches indicated that TiO_2_-NPs have genotoxic properties, including inflammatory cytokines, chromosomal aberrations, gene mutations, gene mutations damage, and deletions in DNA in vitro [10, 11] and in vivo [12]. The toxicity of TiO_2_-NPs was mainly correlated with oxidative stress; therefore, adding nutrients that have antioxidants may be effective for the protection.

Essential oils (EOs) are secondary metabolites found in various aromatic and medicinal plants to protect the host plant from microorganisms [13, 14]. Holy basil (*Ocimum basilicum* L.) is an aromatic herb widely utilized in traditional medicine, and food flavoring. The main constitutes of the holy basil essential oil are terpenoid and phenolic compounds that are ascribed to the therapeutic properties [15, 16]. The basil oil inhibits cholesterol synthesis, enhances digestion, and acts as a chemo-preventive agent besides its pharmacological activities such as the antioxidant, anti-inflammatory, and antimicrobial properties [16–23].

Due to their instability during exposure to light, temperature, oxygen, and pH [20, 24, 25], it was necessary to use encapsulation technology as a modern approach to solve the problems facing the use of EOs in various applications and protect them against chemical and thermal decomposition, increase their bioavailability and water solubility, construction of delivery, and controlling their release [6, 26, 27]. Therefore, this work was designed to determine the bioactive constituents in holy basil essential oil (HBEO), synthesize HBEO nanoemulsion (HBEON), and compare the pharmaceutical effect of HBEO and HBEON against the oxidative stress, genotoxicity, and DNA damage of TiO_2_-NPs in rats.

## Materials and Methods

### Materials

Whey protein isolate (WPI) and titanium tetra isopropoxide were obtained from Sigma-Aldrich (St. Louis, MO, USA). TiO_2_-NPs (average size of 28.0 nm and *ξ*-potential of − 33.97 mV) were biosynthesized as described in our previous work [12] using Titanium tetra isopropoxide and orange peel extract (OPE). The resulting TiO_2_-NPs were dried overnight at 80 °C then were calcined at 600 °C for 4 h [28]. HBEO was provided by the Oil Extraction Unit, National Research Centre (NRC), Dokki, Cairo, Egypt, and was extracted using Clevenger-type apparatus.

### Chemicals and Kits

Kits for Transaminases (ALT and AST), creatinine, urea, cholesterol (Chol), triglycerides (TriG), low- and high-density lipoprotein (LDL, HDL), total protein (TP), and albumin (Alb) were supplied by Randox Co, (Antrim, UK). Kits for nitric oxide (NO), malondialdehyde (MDA), glutathione peroxidase (GPx), catalase (CAT), and superoxide dismutase (SOD) were purchased from Eagle diagnostics (Dallas, TX, USA). Kits for carcinoembryonic antigen (CEA), alpha-fetoprotein (AFP), and tumor necrosis factor-alpha (TNF-α) were provided by BiochemImmuno Systems Co. (Montreal, Canada). Kit for first-strand cDNA synthesis was obtained from iNtRON Biotechnology (Seoul, Korea). SYBR® Premix Ex TaqTM kit was supplied by TaKaRa Biotech. Co. Ltd. (Shiga, Japan). TRIzol® reagent and RNAse-free DNAse kit were purchased from Invitrogen (Germany).

### GC–MS Analysis of HBEO

The analysis of HEBO was conducted using a GC–MS (Hewlett-Packard model 5890) with a flame ionization detector (FID) and DB-5 fused silica capillary column (60 m × 0.32 mm) according to El-Nekeety et al. [29]. The retention indices (Kovats index) of the volatile compounds were calculated as described by Adams [30] using the hydrocarbons as reference (C7-C20, Aldrich Co.).

### Synthesis of HBEON

WPI was used as the wall for the preparation of HBEON according to our previous report [6]. In brief, the WPI solution (10%) was prepared using distilled water, stirred for 1 h, and kept for 12 h at room temperature before emulsification. Tween 80 (80 mg) was used as an emulsifier, and the HBEO was added gradually at a ratio of 2:1 (w/w) with homogenization for 10 min at 20,000 rpm [31]; then, the emulsion was encapsulated by spray drying.

### Characterization of HBEON

Scanning and transmission electron micrographs (TEM and SEM) were done using JEOL JAX-840A and JEOL JEM- 1230 electron micro-analyzers for HBEON. The acquisition of the image was done by Orius 1000 CCD camera (GATAN, Warrendale, PA, USA). HBEON sample was sonicated (30–60 min) immediately to prevent the coalescence of the nanoparticles before the assessment of zeta potential. The average diameter of the synthesized nanoparticles was calculated by zpw 388 version 2.14 nicomp software. However, the zeta potential and size distribution were done by a particle size analyzer (Nano-ZS, Malvern Instruments Ltd., UK).

### Animals and Experimental Setup

Male Sprague–Dawley rats (3 months old, 155 ± 15 g) were provided by the Experimental Animal Facility, Faculty of Veterinary Medicine, Cairo University, Cairo, Egypt. The animals were housed individually in ventilated filter top polycarbonate cages in an artificially illuminated and thermally controlled room (12 h dark/light cycle, 25 ± 1 °C and 25–30% humidity) at the Animal House Lab, Faculty of Pharmacy, Cairo University, and were given ad libitum access to water and rodent chow diet. All animals were left for 1 week as an acclimatization period before starting the experiment, and all the procedures used in this experiment have complied with the guidelines of the National Institute of Health (NIH publication 86–23 revised 1985), and the protocol was approved by the Research Ethics Committee of Faculty of Pharmacy, Cairo University (REC-FOFCU), Egypt. Animals were distributed into 6 groups (10 rats/group) and treated daily by the oral gavage for 21 days as follows: (1) the untreated control group, (2) the HBEO-treated group (5 mg/kg bw), (3) HBEON-treated group (5 mg/kg b.w), (4) the animals that received TiO_2_-NPs (50 mg/kg b.w), and (5, 6) the animals that received TiO_2_-NPs plus HBEO or HBEON. After the last dose, animals have fasted for 12 h; then, samples of blood were withdrawn under isoflurane anesthesia through the retro-orbital venous plexus. The sera were harvested by cooling centrifuging (4 °C) at 3000 rpm for 10 min then kept at − 20 °C until the analysis. These sera were used for the assay of liver and kidney indices, lipid profile, and cytokines (TNF-α, CEA, and AFP) according to the kit’s instructions. Thereafter, all rats were euthanized; liver and kidney samples were weighed homogenized in a phosphate buffer (pH 7.4), and centrifuged (1700 rpm and 4 °C for 10 min). The supernatant was used for NO, MDA, SOD, CAT, and GPx assays [32]. Other liver samples were kept in liquid nitrogen − 80 °C for genetic analysis. However, other liver samples from each animal were fixed in formal saline (10%), dehydrated by a graded series of alcohol, cleaned in xylene, embedded in paraffin wax, and sliced at 5 μm thickness. The sections were stained with hematoxylin and eosin stains for histopathological investigation using a light microscope [33].

### Gene Expression Analysis

#### Total RNA IIsolation

The total genomic RNA in liver samples was isolated using TRIzol® reagent, and the RNA pellets were stored in DEPC-treated water. These pellets were treated with an RNAse-free DNAse kit to digest the potential DNA residues, and the RNA aliquots were stored at − 20 °C until use for the reverse transcription [34].

#### Reverse Transcription Reaction

A copy of cDNA from the liver tissues was synthesized using First Strand cDNA Synthesis Kit via the reverse transcription reaction (RT). The program of RT reaction was 25 °C for 10 min, 1 h at 42 °C then 5 min at 95 °C, and was applied to obtain the cDNA copy of the hepatic genome. The tubes of a reaction containing the cDNA copy were then collected on ice for cDNA amplification [34].

#### Quantitative RT-PCR

SYBR® Premix Ex TaqTM kit was utilized to assay the qRT-PCR analyses for the synthesized cDNA copies of hepatic tissue, and the melting curve profile was performed for each reaction. The specific primers were selected according to the published sequences of Gen Bank. Sequences of caspase-3, Bax, Bcl-2, TNF-α, P53, glyceraldehyde 3-phosphate dehydrogenase (GAPDH) primers, and the annealing temperature used for RT-PCR are shown in Table 1. The housekeeping gene expression was utilized to normalize the quantitative values of the target genes. The 2 − ΔΔCT method was applied for the determination of the quantitative values of the specific genes to GAPDH, and the relative quantification of the target gene to the reference was calculated using the following equations:

$$\begin{array}{lll}{\mathrm{{\Delta}C}}_{\mathrm T(\mathrm{test})}={\mathrm{CT}}_{(\mathrm{target},\;\mathrm{test})}-{\mathrm T}_{(\mathrm{reference},\;\mathrm{test})}\\\mathrm{{\Delta}CT}\;(\mathrm{calibrator})={\mathrm{CT}}_{(\mathrm{target},\;\mathrm{calibrator})}-{\mathrm{CT}}_{(\mathrm{reference},\;\mathrm{calibrator})}\\\mathrm{{\Delta}{\Delta}CT}={\mathrm{{\Delta}C}}_{\mathrm T(\mathrm{Test})}-{\mathrm{{\Delta}C}}_{\mathrm T(\mathrm{calibrator}).}\end{array}$$

**Table 1:** Primer sequences used for real-time PCR

Gene	**Nucleotide sequence 5′–3′**	**Accession no**	Product size (bp)	Annealing (°C)	**References**
Bax	AGGATGATTGCTGATGTGGATAC CACAAAGATGGTCACTGTCTGC	NM_017059.2	300	60	[110]
Caspase-3	AAATTCAAGGGACGGGTCAT ATTGACACAATACACGGGATCTGT	NM_012922.2	652	55	[111]
Bcl-2	GCTACGAGTGGGATACTGGAGA AGTCATCCACAGAGCGATGTT	NM_016993.2	446	60	[112]
P53	GCACAAACACGCACCTCAAAGC CTTGCATTCTGGGACAGCCAAG	NM_030989.3	489 bp	58	[113]
GAPDH	CAAGGTCATCCATGACAACTTTG GTCCACCACCCTGTTGCTGTAG	NM_017008.4	496 bp	58	[50]

#### DNA Fragmentation Assay

DNA fragmentation was determined to evaluate apoptosis following the procedure of Perandones et al. [35]. In brief, 10–20 mg of hepatic tissue were ground in 400-μl hypotonic lysis buffer (1 mM EDTA, 10 mM Tris base, and 0.2% Triton X-100), then centrifuged at 10,000 rpm and 4 °C for 15 min. The supernatant of each sample was divided into two halves, the first was used for gel electrophoresis, and the second was used together with the pellets for the quantification of fragmented DNA by the diphenylamine. The blue color was developed and quantified at 578 nm, and the DNA fragmentation percentage in the sample was calculated using the formula (1):

1 $$\%\;DNA\;fragmentation=\;\left(O.D\;Superna\tan t/\;O.\;D\;Superna\tan t\;+\;O.D\;Pellet\right)\;\times\;100$$

#### Single-Cell Gel Electrophoresis (comet) Assay

The comet assay was done as described in detail by Fahmy et al. [36]. A total of 50 cells were analyzed per animal using automatic comet score™ software (TriTek Corp, version 2.0.0.0, Sumerduck, VA 22,742, USA). Tail DNA percentage (% Tail DNA) and Olive tail moment (OTM) were used as indicators for DNA damage and were expressed in arbitrary units (A.U).

### Statistical Analysis

All data were statistically analyzed using computerized software SPSS (Statistical Package of Social Science, version 20, Armonk, New York: IBM Corp). The one-way analysis of variance (ANOVA) followed by Duncan’s multiple comparisons test was applied to measure the degree of significance at *p* < 0.05.

## Results

**Fig. 1:**
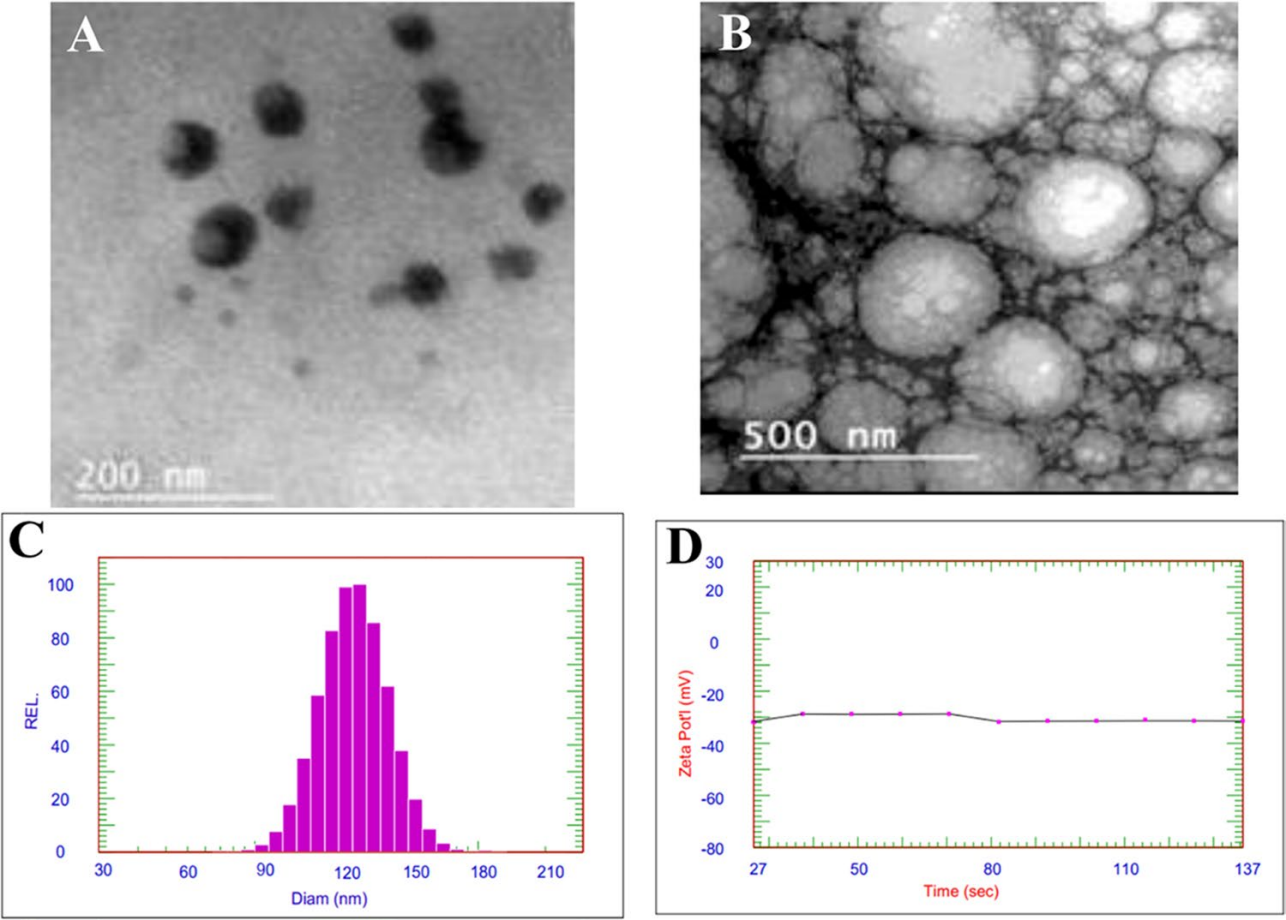
**A** TEM image of HBEON, **B** SEM image of HBEON, **C** DLS analysis showing the size distribution of HBEON, and **D** ZetaSizer chromatogram showing the zeta potential of HBEON

The GC–MS analysis showed the identification of 55 compounds in HBEO represented 98.8% of the oil and the majority belongs to terpene, phenylpropanoids, sesquiterpenoids, and terpene alcohol (Table 2). Five major compounds represented 76.9% including linalool, methyl chavicol, γ-muurolene, β-elemene, and aciphyllene, and were found in concentrations of 53.9, 12.63, 3.7, 3.47, and 3.2%, respectively. However, the other fifty compounds were found in concentrations of less than 2%. The SEM and TEM image of HBEON showed a semi-rounded shape particles (Fig. 1A and B) with an average size of 120 ± 4.5 nm (Fig. 1C) and − 28 ± 1.3 mV zeta potential (Fig. 1D).

**Table 2:** GC/MS analysis of the composition of basil essential oil^a^

No	Compound name^b^	RT	Composition, %
1	Santolina triene	10.44	1.4
2	α-pinene	10.56	0.2
3	Camphene	10.62	0.2
4	Sabinene	10.85	0.1
5	β-Pinene	11.32	0.2
6	Myrcene	11.86	0.4
7	o-Cymene	12.86	0.1
8	Limonene	13.29	0.2
9	1,8-Cineole	13.52	0.8
10	(Z)-β-Ocimene	13.77	0.1
11	(E)-β-Ocimene	14.04	1.7
12	cis-Sabinene hydrate	14.67	0.2
13	cis-Linalool oxide	15.02	0.1
14	Terpinolene	15.23	0.1
15	Linalool	16.03	53.9
16	Camphor	16.84	0.4
17	Menthone	17.63	0.4
18	iso-Menthone	18.32	0.2
19	Menthol	18.67	1.3
20	Terpinen-4-ol	18.88	0.1
21	α-Terpineol	19.29	0.1
22	Methyl chavicol	19.74	12.63
23	Geraniol	20.21	1.1
24	Geranial	21.84	0.1
25	Bornyl acetate	22.39	0.2
26	Menthyl acetate	22.69	0.1
27	δ-Elemene	23.95	0.2
28	α-Cubebene	24.52	0.1
29	Eugenol	25.34	0.1
30	α-Copaene	25.56	0.4
31	Geranyl acetate	25.84	0.1
32	β-Elemene	26.14	3.47
33	α-Cedrene	26.56	0.1
34	(E)-Caryophyllene	26.97	0.7
35	β-Gurjunene	27.09	0.1
36	α-*trans*-Bergamotene	27.22	0.7
37	α-Guaiene	27.39	1.8
38	cis-Muurola-3,5-diene	27.74	0.1
39	α-Humulene	27.96	0.6
40	cis-Cadina-1(6),4-diene	28.12	0.5
41	β-Acoradiene	28.33	0.1
42	γ-Muurolene	28.81	3.7
43	β-Selinene	28.94	0.4
44	Aciphyllene	29.31	1.2
45	Aciphyllene	29.61	3.2
46	γ-Cadinene	29.83	1.7
47	δ-Cadinene	29.91	0.4
48	trans-Cadina-1,4-diene	30.19	0.1
49	α-Cadinene	30.48	0.1
50	(E)-Nerolidol	30.96	0.1
51	Spathulenol	31.58	0.1
52	1,10-di-epi-Cubenol	32.58	0.4
53	epi-α-Cadinol	33.51	1.9
54	β-Eudesmol	33.71	0.1
55	Total identified (%)		98.8

^a^Compound identified by GC/MS and/or by comparison of MS and LRI of standard compounds (ST) under the same conditions

^b^The compounds are listed according to their concentration order. Linear retention index relative to n-alkanes (C7-C20) on DB-5 column

The biochemical assays indicated that TiO_2_-NPs disturb the hepatic and renal functions (Table 3) as evidenced by the remarkable elevation (*p* < 0.05) of the transaminase (AST, ALT), D.BIL, T.BIL, urea, uric acid, and creatinine, and the remarkable decrease (*p* < 0.05) in Alb and TP. Animals that received HBEO were comparable to the untreated control in all the biochemical markers of the liver and kidney except uric acid which was below the control value. However, rats treated with HBEON showed a notable decrease (*p* < 0.05) in transaminases (ALT, AST) and creatinine and a remarkable increase (*p* < 0.05) in Alb and TP with no effects on the other parameters. The combined treatment with TiO_2_-NPs and HBEO or HBEON induced a worthy improvement in the liver and kidney indices, and HBEON was more effective than HBEO to restore most of these markers to the control levels.

**Table 3:** Effect of HBEO and HBEON on serum biochemical parameters in rats treated with TiO_2_-NPs

Groups parameter	Control	HBEO	HBEON	TiO_2_-NPs	TiO_2_-NPs + HBEO	TiO_2_-NPs + HBEON
ALT (U/L)	44.23 ± 1.06^a^	44.58 ± 0.56^a^	42.93 ± 1.03^b^	86.41 ± 2.27^c^	62.19 ± 1.81^d^	52.37 ± 0.43^e^
AST (U/L)	145.11 ± 1.38^a^	145.38 ± 0.71^a^	139.17 ± 2.86^b^	204.51 ± 2.38^c^	159.96 ± 1.51^d^	159.71 ± 1.06^d^
Alb (g/dl)	2.84 ± 0.03^a^	2.55 ± 0.10^b^	2.88 ± 0.04^a^	1.14 ± 0.03^c^	2.21 ± 0.07^d^	2.03 ± 0.05^e^
TP (g/dl)	6.26 ± 0.18^a^	6.26 ± 0.10^a^	6.82 ± 0.05^b^	5.22 ± 0.24^c^	5.46 ± 0.09^d^	6.09 ± 0.08^a^
T.BIL (g/dl)	0.89 ± 0.02^a^	0.83 ± 0.02^a^	0.80 ± 0.02^a^	1.54 ± 0.21^a^	1.13 ± 0.02^a^	0.95 ± 0.04^a^
D.BIL (g/dl)	0.19 ± 0.02^a^	0.21 ± 0.01^a^	0.22 ± 0.01^a^	0.38 ± 0.02^c^	0.30 ± 0.01^d^	0.28 ± 0.02^d^
Creatinine (g/dl)	1.14 ± 0.03^a^	0.85 ± 0.04^ab^	0.86 ± 0.03^b^	2.52 ± 0.11^c^	1.94 ± 0.02^d^	1.81 ± 0.04^e^
Uric acid (mg/dl)	2.26 ± 0.21^a^	1.46 ± 0.07^b^	1.46 ± 0.06^b^	4.17 ± 0.24^c^	2.59 ± 0.09^d^	2.47 ± 0.10^e^
Urea (mg/dl)	56.21 ± 1.38^a^	53.44 ± 0.41^a^	52.31 ± 0.43^a^	81.32 ± 1.51^b^	70.32 ± 0.78^c^	59.85 ± 0.57^d^

Within each column, means superscript with different letters are significantly different (*p* < 005)

TiO_2_-NPs also disturbed the lipid profile (Table 4) as a manifestation of the remarkable increase (*P* < 0.05) in TriG, Chol, and LDL and the decrease in HDL. Administration of HBEO alone decreased Chol, TriG, and LDL but did not affect HDL. However, administration of HBEON decreased Chol and TriG but did not affect LDL or HDL. Co-administration of TiO_2_-NPs plus HBEO normalized Chol and improved significantly (*P* < 0.05) TriG, LDL, and HDL compared to TiO_2_-NPs. More improvement in these indices towards the control values was observed in the group that received HBEON plus TiO_2_-NPs (Table 4).

**Table 4:** Effect of HBEO and HEBEON on serum lipid profile in rats treated with TiO_2_-NPs

Parameter Groups	Chol (mg/dl)	TriG (mg/dl)	LDL (mg/dl)	HDL (mg/dl)
Control	68.44 ± 1.24^a^	87.41 ± 2.66^a^	35.24 ± 1.38^a^	33.46 ± 2.51^a^
HBEO	64.61 ± 0.93^b^	81.32 ± 1.12^b^	32.69 ± 0.70^b^	34.63 ± 0.41^a^
HEBEON	63.22 ± 1.02^b^	79.58 ± 0.45^c^	34.25 ± 0.61^a^	35.49 ± 0.52^a^
TiO_2_-NPs	87.68 ± 2.52^c^	124.42 ± 2.58^d^	66.57 ± 2.47^c^	22.52 ± 1.27^b^
TiO_2_-NPs + HBEO	69.94 ± 0.52^a^	102.10 ± 1.02^e^	52.15 ± 0.75^d^	26.17 ± 0.86^c^
TiO_2_-NPs + HEBEON	60.82 ± 0.86^d^	101.81 ± 1.01^e^	49.30 ± 0.75^e^	26.67 ± 0.48^c^

Within each column, means superscript with different letters are significantly different (*p* <0.05)

**Fig. 2:**
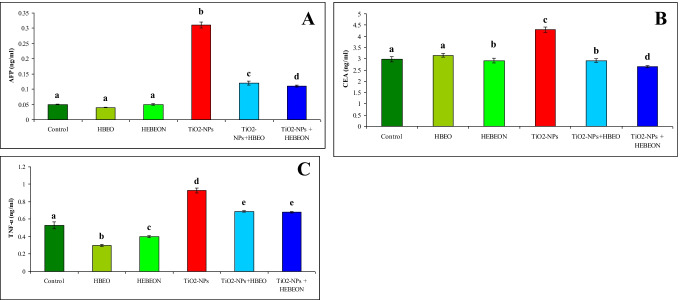
Effect of HBEO and HEBEON on serum AFP (**A**), CEA (**B**), and TNF-α (**C**) in rats treated with TiO_2_-NPs. Superscripts with different letters in each column are significantly different at *p *< 0.05

The effect of TiO_2_-NPs alone or plus HBEO or HBEON on serum cytokines (Fig. 2) indicated that TiO_2_-NPs induced a marked elevation (*p* < 0.05) in AFP, CEA, and TNF-α. Administration of HBEO or HBEON induced a marked reduction (*p* < 0.05) in TNF-α but did not affect AFP or CEA. Both HBEO and HBEON induced a considerable improvement (*p* < 0.05) in the tested cytokines in the animal that received the combined treatment with TiO_2_-NPs towards the normal control level although their values were still higher compared with the untreated control rats.

**Fig. 3:**
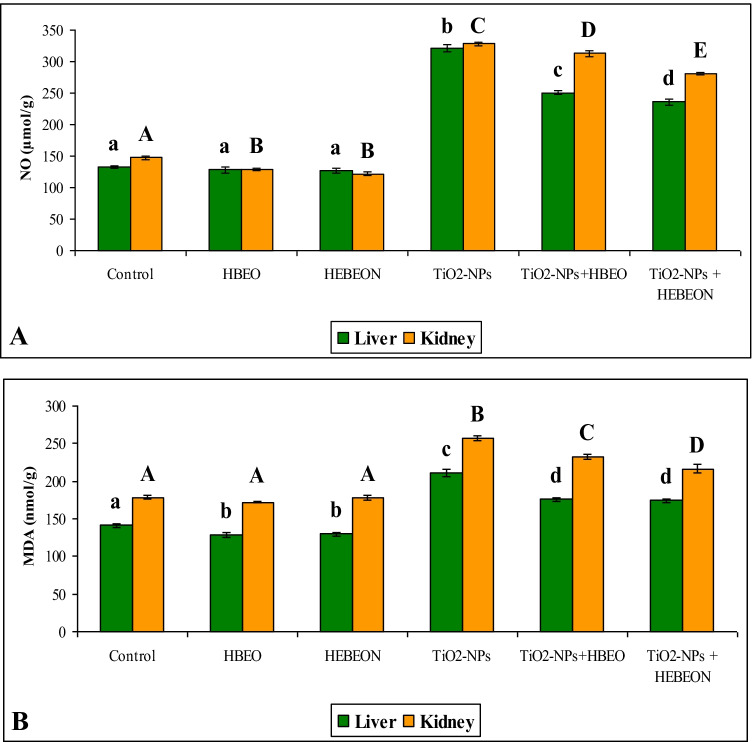
Effect of HBEO and HBEON on hepatic and renal NO (**A**) and MDA (**B**). Superscripts with different letters in each column (small letters for liver and capital letters for kidney) are significantly different at *p *< 0.05

The current results also indicated that TiO_2_-NPs increased hepatic and renal NO levels compared with those in the untreated control group (Fig. 3A). HBEO and HBEON decreased the renal NO but did not induce a marked effect on its hepatic level. Co-administration of TiO_2_-NPs plus HBEO or HBEON significantly improved (*P* < 0.05) NO in both organs, and HBEON was more effective compared to HBEO. Additionally, TiO_2_-NPs also increased the hepatic and renal MDA compared with those in the negative control group (Fig. 3B). HBEO and HBEON decreased significantly (*p* < 0.05) the hepatic MDA but did not cause any marked changes in renal MDA level. The combined treatment with TiO_2_-NPs plus HBEO or HBEON improved significantly (*p* < 0.05) MDA in both liver and kidney, although it was still higher than the level of control in both organs.

The data listed in Table 5 showed that TiO_2_-NPs significantly (*p* < 0.05) reduced the hepatic and renal antioxidant enzymes activity. HBEO alone increased the activity of hepatic and renal GPx and renal CAT but no significant (*P* < 0.05) effect was noticed in hepatic CAT or SOD in these organs. Co-treatment with TiO_2_-NPs plus HBEO or HBEON improved these antioxidant enzymes in both organs, and HBEON was more effective than HBEO.

**Table 5:** Effect of HBEO and HBEON on hepatic and renal antioxidant enzymes in rats treated with TiO_2_-NPs

Parameter groups	GPx (U/g)		CAT (mU/g)		SOD (U/g)	
	Liver	Kidney	Liver	Kidney	Liver	Kidney
Control	36.45 ± 1.07^a^	31.30 ± 0.98^a^	6.56 ± 0.08^a^	7.88 ± 0.15^a^	28.14 ± 0.65a^a^	28.19 ± 0.50^a^
HBEO	39.49 ± 2.24^b^	33.49 ± 1.13^b^	7.02 ± 0.10^a^	8.05 ± 0.08^b^	29.89 ± 0.77^a^	29.93 ± 0.42^a^
HEBEON	41.25 ± 2.51^b^	35.63 ± 0.45^c^	7.16 ± 0.05^a^	9.22 ± 0.26^c^	29.87 ± 0.49^a^	28.43 ± 1.04^a^
TiO_2_-NPs	21.43 ± 0.55^c^	12.02 ± 0.57^d^	2.87 ± 0.15^b^	3.46 ± 0.15^d^	14.24 ± 0.15^b^	15.02 ± 0.25^b^
TiO_2_-NPs + HBEO	27.41 ± 0.78^d^	17.38 ± 0.24^e^	4.33 ± 0.09^c^	5.31 ± 0.05^e^	17.73 ± 0.34^c^	18.86 ± 0.34^c^
TiO_2_-NPs + HBEON	30.82 ± 0.46^f^	23.68 ± 0.54^f^	5.01 ± 0.07^d^	5.54 ± 0.08^e^	21.45 ± 0.54^d^	20.73 ± 0.27^d^

Within each column, means superscript with different letters are significantly different (*p*<0.05)

**Fig. 4:**
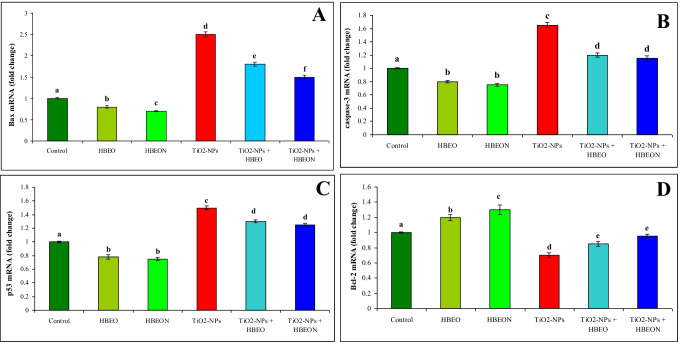
Effect of HBEO and HBEON on relative expression of Bax (**A**), caspase-3 (**B**), and p53 (**C**) and Bcl-2 gene in liver of rats treated with TiO_2_-NPs. Analyses were performed in triplicate. Data are the mean ± SE of three different liver samples in same group. Superscripts with different letters in each column are significantly different at *p* < 0.05

The mechanisms of prevention of HBEO and HBEON against hepatic apoptosis induced by TiO_2_-NPs were further evaluated in this study. Pro-apoptotic gene Bax and anti-apoptotic gene Bcl-2 were examined using qRT-PCR. Administration of HBEO or HBEON alone decreased the mRNA expression of Bax (Fig. 4A), caspase-3 (Fig. 4B), and P53 (Fig. 4C); however, these treatments increased the expression of Bcl-2 mRNA (Fig. 4D). TiO_2_-NPs upregulated the mRNA expression Bax (2.5-fold increase), caspase-3 (1.65-fold increase), and p53 (1.5-fold increase) and downregulated the expression of Bcl-2 mRNA (0.7-fold decrease) compared with the untreated control group. Co-treatment with TiO_2_-NPs plus HBEO or HBEON improved mRNA expression of these genes, and this improvement was more noticeable in the group that received HBEON. The relative values were a 1.4- and 1.6-fold decrease for Bax, a 1.4- and 1.43-fold decrease for caspase-3, and a 1.1- and 1.2-fold decrease for p53; however, the relative value for Bcl-2 was 1.2- and 1.4-fold increase in the groups received TiO2-NPs plus HBEO or HBEON, respectively.

**Fig. 5:**
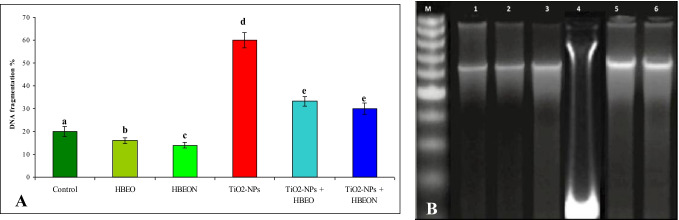
Effect of HBEO or HBEON alone or in combination with TiO_2_-NPs on hepatic **A** DNA fragmentation percentage and **B** DNA fragmentation analysis: 1.5% of agarose gel electrophoresis of DNA samples extracted from liver tissues of different groups. Lane M: 100 bp size marker, Lane 1: control group, Lane 2: HBEO, Lane 3: HBEON, Lane 4: TiO_2_-NPs, Lane 5: TiO_2_-NPs plus HBEO, and Lane 6: TiO_2_-NPs plus HBEON

The current results showed that the percentage of DNA fragmentation in the hepatic tissue of TiO_2_-NP-treated rats was significantly increased (*p*< 0.05) in comparison with the negative control group. Administration of HBEO or HBEON decreased significantly DNA fragmentation percent, and this decrease was apparent in the group that received HBEON. On the other hand, co-administration with TiO_2_-NPs plus HBEO or HBEON showed a remarkable decrease in DNA fragmentation percent and HBEON was more effective than HBEO (Fig. 5A, B).

**Fig. 6:**
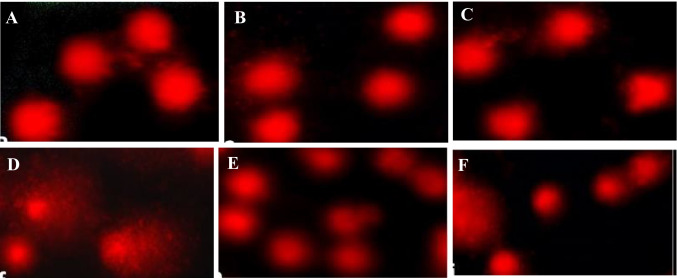
Alkaline comet assay for DNA damage in liver of **A** control rats, **B** HBEO-treated rats, **C** HBEON-treated rats, **D** TiO_2_-NP-treated rats, **E** TiO_2_-NPs + HBEO-treated rats, and **F** TiO_2_-NPs + HBEON-treated rats (original magnification 400 ×)

The data in Table 6 and Fig. 6 showed the effect of different treatments on the level of DNA breakages in the liver using the comet assay. Treatment with HBEO or HBEON alone did not increase the percentage of tail DNA in rat liver (9.83% and 9.75% for HBEO and HBEON, respectively, versus 9.77% for control). Further, the OTM values did not change significantly (*p* > 0.05) in the groups treated with HBEO or HBEON (1.13 and 1.12 A. U, respectively) compared to the normal control value (1.12 A.U). Treatment with TiO_2_-NPs increased tail DNA percentage (18.74%) and OTM (3.57 A. U) compared with the normal control values (1.12 A. U). Additionally, co-administration of HEBO or HBEON plus TiO_2_-NPs induce a significant decrease in tail DNA percentage and OTM compared to TiO_2_-NPs alone-treated group and this reduction was more noticeable in the group treated with TiO_2_-NPs plus HBEON than that in TiO_2_NPs plus HEBO-treated group.

**Table 6:** Inhibitory activity of HBEO and HBEON on DNA damage induced by TiO_2_-NPs in rat liver using comet assay (mean ± S.E)

Treatment groups	% Tail DNA	OTM (A.U)
Control	9.77 ± 1.24^a^	1.12 ± 0.02^a^
HBEO	9.83 ± 0.08^a^	1.13 ± 0.02^a^
HEBEON	9.75 ± 0.53^a^	1.12 ± 0.06^a^
TiO_2_-NPs	18.74 ± 1.77^b^	3.57 ± 0.14^b^
TiO_2_-NPs + HBEO	12.40 ± 0.27^c^	1.55 ± 0.03^c^
TiO_2_-NPs + HBEON	11.23 ± 0.37^d^	1.12 ± 0.11^a^

The values superscript with different letters in each column are significantly different from one another as calculated by ANOVA (*P* < 0.05)

**Fig. 7:**
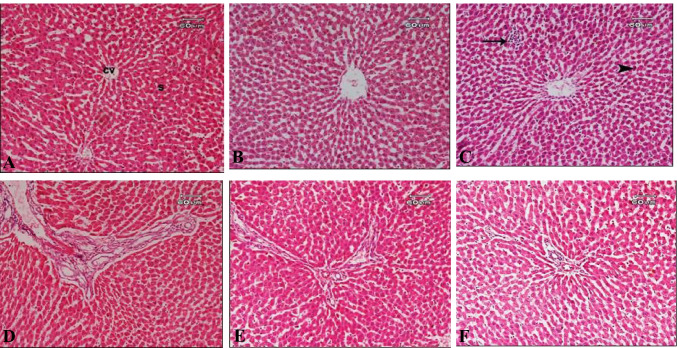
Photomicrograph of the liver sections of **A** control rats showing normal structure of hepatic lobule, central vain (cv), and blood sinusoid (s); **B** rats treated with HBEO showing normal hepatic structure; **C** rats treated with HBEON showing the normal structure of hepatic tissue while, nodule of aggregation of inflammatory cells (arrow) were noted, sings of nuclear degeneration in the form of necrosis, pyknosis (arrow head), and karyolysis were noted; **D** rats treated with TiO_2_-NPs showing marked dilatation of the portal tract, proliferation of the bile ducts and necrosis in their epithelial cells, and fibrosis (formation of fibrous septa) could be observed; **E** rats treated with TiO_2_-NPs plus HBEO showing some improvement represented in minimal fibrous tissue and little diffusion of inflammatory cell infiltration; and **F** rats treated with TiO_2_-NPs plus HBEON showing improvement in pathological changes in the form of diminution in fibrosis, and weak dilation in portal tract, mild cellular infiltration and hypertrophied of kupffer cell

The histological examination of the control liver sections showed the normal structure of the hepatic lobule, central vein, and blood sinusoid (Fig. 7A). The sections of the liver from animals that received HBEO revealed normal hepatic structure, although some nuclei appear small in size and some of them showed signs of degeneration such as necrosis, karyolysis, and pyknosis (Fig. 7B). The liver sections of the rats that were treated with HBEON showed normal structure, while nodules of aggregation of inflammatory cells and signs of nuclear degeneration in the form of necrosis, pyknosis, and karyolysis were noted (Fig. 7C). The liver sections of rats that were treated with TiO_2_-NPs showed marked dilatation of the portal tract, the proliferation of bile ducts, necrosis in their epithelial cells, and fibrosis (formation of fibrous septa) could be also observed (Fig. 7D). Liver sections of rats that received TiO_2_-NPs plus HBEO showed some improvement represented in reduction of fibrous tissue, mild cellular infiltration around the dilated portal area, and mildly dilated bile duct; however, signs of degeneration in the form of pyknosis, karyolysis, and necrosis, as well as vacuolar degeneration, were still present (Fig. 7E). On the other hand, animals that were treated with TiO_2_-NPs plus HBEON showed improvement in the pathological alterations in the form of diminution in fibrosis, weak dilation in the portal tract, minute cytoplasmic vacuoles, mild cellular infiltration, and hypertrophied of Kupffer cells (Fig. 7F).

## Discussion

In this study, the GC–MS identified 55 compounds in HBEO that constituted 98.8% of the oil and belong to terpene, phenylpropanoids, sesquiterpenoids, and terpene alcohol. The major identified compounds were linalool and methyl chavicol followed by γ-muurolene, β-elemene, and aciphyllene. Previous studies reported that linalool was the major compound identified in HBEO [37, 38]; however, other studies reported that methyl eugenol was the major compound followed by methyl chavicol [39], and others showed that methyl chavicol followed by linalool [40]. In this concern, Ahmed et al. [41] and Diniz do Nascimento et al. [42] suggested that the chemical compounds of the oils differ according to several factors including the plant variety, agriculture practices, and geographical origin. The HOBEN was synthesized successfully by incorporating WPI, and the resulted emulsion showed a smooth and semi-round shape with an average size and a *ζ*-potential of 120 nm and − 28 mV, respectively. These results suggested that WPI increased the coalescence of the droplets [43], and also, the smooth shape and the uniform size distribution confirmed that WPI acted as a wall material for the oil droplets [44, 45]. Roger et al. [46] indicated that the particles’ size and the properties of their surface have a vital role in the uptake of the nanoparticles by the cells and the favorable size is 50–300 nm compared with the other sizes, although the size < than 100 nm showed unique and novel functional properties. Moreover, these authors reported that size has a vital role in the distribution, pharmacokinetics, and clearance of nanoparticles. Additionally, *ζ*-potential also has a critical effect on the distribution and stability of the droplets [47]. In our study, the negative *ζ*-potential of HBEON is ascribed to the negative charge of the carboxylate group as it is the functional charge of the WPI globule [44].

The in vivo study was performed to estimate the protective role of HBEON compared to HBEO against TiO_2_-NP-induced oxidative damage and genotoxicity in rats. The doses of TiO_2_-NPs and the oils were selected based on our previous reports [6,29, respectively]. Administration of TiO_2_-NPs displayed severe disturbances in the biochemical indices, the oxidant/antioxidant parameters, serum cytokines, gene expression, DNA damage, and the histological construction of the hepatic tissues. The increase of AST and ALT in the TiO_2_-NPs-treated group indicated that these nanoparticles induced injury and damage to the hepatocytes resulting in the release of these enzymes into the circulation [48, 49], and the decrease in Alb and TP in this group also confirmed the hepatic damage and/or the kidney dysfunction [50]. Additionally, the increase in creatinine, uric acid, and urea indicated nephrotoxicity and glomerular injury [51, 52]. The increase in Chol, TriG, and LDL and the decrease in HDL after TiO_2_-NP administration indicated that these nanoparticles disturbed the lipid metabolism possibly through the alteration of lipoprotein lipase or the capability of removing or transferring the fractions of lipids [6, 53]. It is known that the elevation in TriG and Chol levels are correlated with some metabolic syndromes and cardiovascular diseases; hence, these results suggest that TiO_2_ could aggravate cardiovascular diseases [54–56].

It was documented that TiO_2_-NPs induce oxidative damage and disturb the balance between oxidant/antioxidant (redox balance) in the body [12, 55, 57]. This fact confirms the present results as the animals in the TiO_2_-NPs group exhibited a significant elevation in MDA, NO, and the inflammatory cytokines (TNF-α, AFP, and CEA) besides a decrease in the activity of the antioxidant enzymes (GPx, SOD, and CAT). Therefore, TiO_2_-NPs enhance ROS generation [57] which injures the macromolecules mainly DNA, carbohydrates, proteins, and lipids [6, 50]. In addition, ROS increases the peroxidation of lipids and disturbs the cell membrane structure and the pivotal function of the cells [58]. The generation of hydroxyl radicals (•OH) is the main factor in the oxidative damage induced by TiO_2_-NPs [59], and the accumulation of MDA besides the decrease in antioxidant enzymes may lead to the apoptosis of cells [6, 12]. Furthermore, Nrf2 as the principal regulator for different antioxidant gene expressions is increased during the overproduction of ROS which resulted in further damage to DNA and enhances the risk of cancer [60]. Moreover, when SOD decrease, hydrogen peroxide (H_2_O_2_) accumulates in the liver and kidney leading to the inhibition of CAT [61], the enzyme responsible for the converting of H_2_O_2_ to H_2_O and O_2_, and hence, it prevents oxidative damage to these organs [62].

TiO_2_-NP administration disturbs the gene expression of Bax, caspase-3, p53, and Bcl-2. Animals in this group exhibited a significant upregulation of Bax, caspase-3, and p53 mRNA expression and a significant downregulation of Bcl-2. These results along with the disturbances in cytokines and immune function indicated that the generation of ROS after TiO_2_-NP exposure activates several receptors resulting in the activation of signaling pathways to decrease the antioxidant via ROS formation [3]. The exposure to excess ROS reduced the mitochondrial membrane potential leading to apoptotic cell death and immunotoxicity through the imbalance of immune redox [3, 5, 63]. Moreover, Bcl-2 is a protein located on the mitochondrial surface to prevent cytochrome c release, but Bax induces punching of the mitochondrial membrane holes to prevent the leaking out of cytochrome c [64, 65], hence, the imbalance between Bcl-2 and Bax activate caspase-dependent apoptotic pathway [66]. Additionally, the increase in Bax mRNA expression may counteract the action of p53 on apoptosis [67]. Animals that received TiO_2_-NPs also showed an increase in the DNA fragmentation percentage in the hepatic tissue which confirms the generation of hydroxyl radical, the major destructive species that enhance DNA damage [59].

The comet assay showed that TiO_2_-NPs increased comet tail formation in the hepatic cells and suggesting that these particles induced single- or double-strand breaks, dysregulation of the cell cycle checkpoints, and DNA-adduct formation [68, 69]. Two main mechanisms were suggested for these consequences as the primary and secondary genotoxicities in the absence and presence of inflammation [70]. According to Jin et al. [71], genotoxicity takes place when TiO_2_-NPs react directly with the DNA molecules or indirectly react with the nuclear proteins. The primary genotoxicity occurred owing to ROS generation and the releasing of the toxic metal ions from the soluble TiO_2_-NPs. On the other hand, secondary genotoxicity occurs through the immune cells which can generate excess ROS and stimulate the release of pro-inflammatory cytokines, thus attacking DNA molecules [72, 73]. Additionally, TiO_2_-NPs stimulate sister chromatid exchange, micronuclei formation, and comet tail in the lymphocytes of peripheral blood in humans [10, 74]. In this concern, Sycheva et al. [75] reported that TiO_2_-NP administration induced the comet tail formation in the liver and bone marrow of mice; meanwhile, the intra-peritoneal injection produces DNA break and DNA adducts [76] as well as increases the cell micronuclei in bone marrow [77].

The histological study revealed that TiO_2_-NP administration induced a marked dilatation of the portal tract, a proliferation of bile ducts and necrosis in their epithelial cells, and fibrosis. These pathological changes were similar to those reported in previous research [6, 78, 79] which showed that treatment with TiO_2_-NPs affects the histological structure of the liver via oxidative damage which was more localized around the central vein. Pialoux et al. [80] suggested a strong linkage between tissue anoxia and oxidative damage in several organs. Additionally, Kupffer cells in the liver are the most impacted cells by oxidative damage due to their location nearby the portal area in the liver tissue [81].

It is clear now that the toxicity of TiO_2_-NPs is particularly due to the excess ROS generation leading to oxidative damage. Hence, antioxidant supplementation probably is valuable in the protection against this oxidative damage. HBEO is known as an affluent source of many bioactive compounds which possess a potent antioxidant. The high level of linalool and methyl chavicol gives this essential oil the advantage to protect against oxidative DNA damage [82, 83]. Moreover, the emulsifying technology manages the controlled release of the bioactive constituents and enhances their bioavailability and stability [50]. In the current study, we evaluated the potential protective role of HBEON compared with HBEO in animals treated with TiO_2_-NPs. Administration of HBEO or HBEON induced positive effects on most of the tested parameters and did not induce any toxic effects. Moreover, both agents induced remarkable improvement in the liver and kidney indices, lipid profile, serum cytokines, and oxidant/antioxidant indices in rats that received TiO_2_-NPs which is due to the potent antioxidant activity of linalool [41, 84], and the pro-oxidant activity which prevents DNA damage and suppression of ROS generation [85]. The previous studies reported that linalool diminishes TNF-α and IL-6 and prevents IkBa protein phosphorylation, p38, c-Jun terminal kinase, and the extracellular signal-regulated kinase [86]. In addition, linalool showed beneficial effects in the attenuation of the expression of NF-kB and TGF-b1 in the kidney of diabetic subjects [87] and prevents the releasing of pro-inflammatory factors, and inhibits the caspase-3, and caspase-8 expression, and the inflammatory response through the suppression of NF-kB [88, 89].

The second protective property of HBEO is due to the methyl chavicol as the second main compound in the oil belonging to the phenylpropanoids class [90] and can block the voltage-activated sodium channels [91]. This compound also has anti-inflammatory activity by inhibiting leukocyte migration and the stimulation of macrophages’ phagocytosis [92]. Moreover, γ-γ-Muurolene as the third principle compound in HBEO is known to possess antioxidant and anti-inflammatory effects [93, 94]. β-elemente is also considered the fourth major compound in HBEO and can regulate oxidative stress and different inflammatory cytokines such as IFN, TNF-α, IL-6/10, and TGF-β in the in vivo and in vitro studies [95]. This compound also induces the apoptosis of tumor cells, and inhibits the P21-activated kinase1 (PAK1) signaling pathway [96, 97] and is used for the treatment of cancer in different organs including the liver [98], stomach [97], lung [99], brain [100], ovary [101], and breast [102].

Additionally, the minor phenolic compounds in the oil have high antioxidant properties and reduce the level of LDL, TriG, and Chol in plasma besides their free radical scavenger activity [103]. Additionally, both HBEO and HBEON increased SOD and CAT, the major hepato-protective endogenous enzymes [104]. Taken together, the mechanisms of antioxidant properties of HBEO and HBEON could be due to the ROS scavenging activity, the iron chelation which initiates the radical reactions, and the inhibition of different enzymes accountable for ROS generation [105], the interference of antioxidants with xenobiotic-metabolizing enzymes which block the activated mutagens/carcinogens, and modulates DNA repair along with the regulation of the mRNA gene expressions [106]. Therefore, these mechanisms are very important for the antioxidant, anticarcinogenic, and antimutagenic properties of the oils [107]. These results also showed that HBEON was more effective than HBEO which may be due to the antioxidant effect of WPI used in the preparation of emulsion. WPI is rich in certain amino acids which are known as potent antioxidants such as cysteine, bovine serum albumin, β-lactoglobulin, and α-lactoglobulin [108], and showed potent hepatoprotective against CCl_4_-induced liver damage [26, 109].

## Conclusion

A total of 55 compounds were identified in HBEO representing 98.8% of the oil. The major compound was linalool followed by Methyl chavicol, γ-Muurolene, β-elemene, and Aciphyllene. TiO_2_-NP administration to rats induced severe oxidative damage in the liver and kidney, increased serum cytokines and DNA fragmentation, disturbed apoptotic gene expression, and histological alteration in the liver. Both HBEO and HBEON with average particles size and *ζ*-potential were 120 ± 4.5 nm and − 28 ± 1.3 mV were safe and succeeded to induce potent protection against TiO_2_-NPs; however, HBEON was more effective than HBEO. This effect suggested that the encapsulation of HBEO using WPI enhances the protective role of the bioactive compounds, controls their release, and increased the antioxidant activity. Therefore, HBEON is a good tool for the protection against oxidative damage; disturbances in biochemical parameters, gene expression, DNA damage, and the histological changes result from the exposure to TiO_2_-NPs and may be suitable for the application in medical, food, and pharmaceutical sectors.

## Data Availability

NA.

## References

[1] Bayda S, Adeel M, Tuccinardi T, Cordani M, Rizzolio F (2019). The history of nanoscience and nanotechnology: from chemical-physical applications to nanomedicine. Molecules (Basel, Switzerland).

[2] Hong F, Yu X, Wu N, Yu-Qing Zhang YQ (2017) Progress of in vivo studies on the systemic toxicities induced by titanium dioxide nanoparticles. Toxicol Res 6:115–133 10.1039/c6tx00338a PMC6061230 30090482

[3] Baranowska-Wójcik E, Szwajgier D, Oleszczuk P, Winiarska-Mieczan A (2020). Effects of titanium dioxide nanoparticles exposure on human health-a review. Biol Trace Elem Res.

[4] Kandeil MA, Mohammed ET, Hashem KS, Aleya L, Abdel-Daim MM (2019). Moringa seed extract alleviates titanium oxide nanoparticles (TiO2-NPs) induced cerebral oxidative damage and increases cerebral mitochondrial viability. Environ Sci Pollut Res.

[5] Rashid MM, Forte Tavčer P, Tomšič B (2021). Influence of titanium dioxide nanoparticles on human health and the environment. Nanomaterials (Basel).

[6] Abdel-Wahhab MA, El-Nekeety AA, Mohammed HE, Elshafey OI, Abdel-Aziem SH, Hassan NS (2021). Elimination of oxidative stress and genotoxicity of biosynthesized titanium dioxide nanoparticles in rats via supplementation with whey protein-coated thyme essential oil. Environ Sci Pollut Res.

[7] Singh SP, Rahman MF, Murty US, Mahboob M, Grover P (2013). Comparative study of genotoxicity and tissue distribution of nano and micron sized iron oxide in rats after acute oral treatment. Toxicol Appl Pharmacol.

[8] Praphawatvet T, Peters JI, Williams RO (2020). Inhaled nanoparticles—an updated review. Int J Pharm.

[9] Bu Q, Yan G, Deng P, Peng F, Lin H, Xu Y, Cao Z, Zhou T, Xue A, Wang Y, Cen X, Zhao YL (2010). NMR-based metabonomic study of the sub-acute toxicity of titanium dioxide nanoparticles in rats after oral administration. Nanotechnol.

[10] Charles S, Jomini S, Fessard V, Bigorgne-Vizade E, Rousselle C, Michel C (2018). Assessment of the in vitro genotoxicity of TiO_2_ nanoparticles in a regulatory context. Nanotoxicol.

[11] Shukla RK, Sharma V, Pandey AK, Singh S, Sultana S, Dhawan A (2011). ROS-mediated genotoxicity induced by titanium dioxide nanoparticles in human epidermal cells. Toxicol In Vitro.

[12] Salman AS, Al-Shaikh TM, Hamza ZK, El-Nekeety AA, Bawazir SS, Hassan NS, Abdel-Wahhab MA (2021). Matlodextrin-cinnamon essential oil nanoformulation as a potent protective against titanium nanoparticles-induced oxidative stress, genotoxicity, and reproductive disturbances in male mice. Environ Sci Pollut Res.

[13] Maes C, Bouquillon S, Fauconnier ML (2019). Encapsulation of essential oils for the development of biosourced pesticides with controlled release: a review. Molecules.

[14] Mittal RP, Rana A, Jaitak V (2019). Essential oils: an impending substitute of synthetic antimicrobial agents to overcome antimicrobial resistance. Curr Drug Targets.

[15] Chitprasert P, Sutaphanit P (2014). Holy basil (*Ocimum sanctum* Linn.) essential oil delivery to swine gastrointestinal tract using gelatin microcapsules coated with aluminum carboxymethyl cellulose and beeswax. J Agric Food Chem.

[16] Rezzoug M, Bakchiche B, Gherib A, Roberta A, Guido F, Kilinçarslan Ö, Mammadov R, Bardaweel SK (2019) Chemical composition and bioactivity of essential oils and ethanolic extracts of *Ocimum basilicum* L. and *Thymus algeriensis* Boiss and Reut from the Algerian Saharan Atlas. BMC Complement Altern Med 19(1): 146. 10.1186/s12906-019-2556-y 10.1186/s12906-019-2556-y PMC6588939 31227024

[17] Dawood M, El Basuini MF, Zaineldin AI, Yilmaz S, Hasan MT, Ahmadifar E, El Asely AM, Abdel-Latif H, Alagawany M, Abu-Elala NM, Van Doan H, Sewilam H (2021). Antiparasitic and antibacterial functionality of essential oils: an alternative approach for sustainable aquaculture. Pathogens (Basel, Switzerland).

[18] Ebani VV, Nardoni S, Bertelloni F, Pistelli L, Mancianti F (2018). Antimicrobial activity of five essential oils against bacteria and fungi responsible for urinary tract infections. Molecules.

[19] Eftekhar N, Moghimi A, Mohammadian Roshan N, Saadat S, Boskabady MH (2019). Immuno-modulatory and anti-inflammatory effects of hydro-ethanolic extract of *Ocimum basilicum* leaves and its effect on lung pathological changes in an ovalbumin-induced rat model of asthma. BMC Complement Altern Med.

[20] Fitsiou E, Pappa A (2019). Anticancer activity of essential oils and other extracts from aromatic plants grown in Greece. Antioxidants.

[21] Ghosh V, Mukherjee A, Chandrasekaran N (2013). Formulation and characterization of plant essential oil based nanoemulsion: evaluation of its larvicidal activity against *Aedes aegypti*. Asian J Chem.

[22] Majdi C, Pereira C, Dias MI, Calhelha RC, Alves MJ, Rhourri-Frih B, Charrouf Z, Barros L, Amaral JS, Ferreira ICFR (2020) Phytochemical characterization and bioactive properties of cinnamon basil (*Ocimum basilicum* cv. 'Cinnamon') and lemon basil (*Ocimum citriodorum*). Antioxidants (Basel) 9(5): 369. 10.3390/antiox9050369. 10.3390/antiox9050369 PMC7278754 32365570

[23] Shah B, Davidson PM, Zhong Q (2013). Nano dispersed eugenol has improved antimicrobial activity against *Escherichia coli* O157:H7 and *Listeria monocytogenes* in bovine milk. Int J Food Microbiol.

[24] Aguilar-Veloz LM, Calderón-Santoyo M, González YV, Ragazzo-Sánchez JA (2020). Application of essential oils and polyphenols as natural antimicrobial agents in postharvest treatments: Advances and challenges. Food Sci Nutr.

[25] Sandra F, Saidi S, Richard H, Ellen A (2019). Essential oils and their applications—a mini review. Adv Nutr Food Sci.

[26] Abdel-Wahhab MA, El-Nekeety AA, Hassan NS, Gibriel AA, Abdel-Wahhab KG (2018). Encapsulation of cinnamon essential oil in whey protein enhances the protective effect against single or combined sub-chronic toxicity of fumonisin B_1_ and/or aflatoxin B_1_ in rats. Environ Sci Pollu Res.

[27] Lammari N, Louaer O, Meniai AH, Elaissari A (2020). Encapsulation of essential oils via nanoprecipitation process: overview, progress, challenges and prospects. Pharmaceutics.

[28] Rao GK, Ashok CH, Venkateswara Rao K, Shilpa Chakra CH, Rajendar V (2015) synthesis of Tio_2_ nanoparticles from orange fruit waste. Int J Multidiscip Adv Res Trends II(I): 82–90.

[29] El-Nekeety AA, Hassan ME, Hassan RR, Elshafey OI, Hamza ZK, Abdel-Aziem SH, Hassan NS, Abdel-Wahhab MA (2021). Nanoencapsulation of basil essential oil alleviates the oxidative stress, genotoxicity and DNA damage in rats exposed to biosynthesized iron nanoparticles. Heliyon.

[30] Adams RB (2007). Identification of essential oil components by gas chromatography/quadruple mass spectroscopy.

[31] Jinapong N, Suphantharika M, Jammong P (2008). Production of instant soymilk powders by ultrafiltration, spray drying and fluidized bed agglomeration. J Food Eng.

[32] Lin CC, Hsu YF, Lin TC, Hsu FL, Hsu HY (1989). Antioxidant and hepatoprotective activity of Punicalagin and Punicalin on carbon tetrachloride induced liver damage in rats. J Pharm Pharmacol.

[33] Bancroft D, Stevens A, Turmer R (1996) Theory and practice of histological technique, 4^th^ ed. Churchill Living Stone, Edinburgh 36–42.

[34] El-makawy A, Ibrahim FM, Mabrouk DM, Abdel-Aziem SH, Sharaf HA, Ramadan MF (2020). Efficiency of turnip bioactive lipids in treating osteoporosis through activation of Osterix and suppression of Cathepsin K and TNF-α signaling in rats. Environ Sci Pollu Res.

[35] Perandones CE, Illera VA, Peckham D, Stunz LL, Ashman RF (1993) Regulation of apoptosis in vitro in mature murine spleen T cells. J Immunol 151:3521–3529 8376790

[36] Fahmy MA, Diab KA, Abdel-Samie NS, Omara EA, Hassan ZM (2018). Carbon tetrachloride induced hepato/renal toxicity in experimental mice: antioxidant potential of Egyptian *Salvia officinalis L* essential oil. Environ Sci Pollut Res.

[37] Amor G, Sabbah M, Caputo L, Idbella M, De Feo V, Porta R, Fechtali T, Mauriello G (2021). Basil essential oil: composition, antimicrobial properties and microencapsulation to produce active chitosan films for food packaging. Foods (Basel, Switzerland).

[38] Benedec D, Oniga I, Toiu A, Tiperciuc B, Tămaş M, Vârban, ID, Crişan G (2013) GC-MS analysis of the essential oil obtained from *ocimum basilicum* L.“holland” cultivar FARMACIA 61(3): 448–453.

[39] Olugbade TA, Kolipha-Kamara MI, Elusiyan CA, Onawunmi GO, Ogundaini AO (2017). Essential oil chemotypes of three *ocimum* species found in Sierra Leone and Nigeria. Med Aromat Plants.

[40] Ghasemi Pirbalouti A, Malekpoor F, Salimi A (2017). Chemical composition and yield of essential oil from two Iranian species of basil (*Ocimum ciliatum* and *Ocimum basilicum*). Trends Phytochem Res.

[41] Ahmed AF, Attia FAK, Liu Z, Li C, Wei J, Wenyi Kang W (2019) Antioxidant activity and total phenolic content of essential oils and extracts of sweet basil (*Ocimum basilicum* L.) plants. Food Sci Hum Well 8(3): 299–230.

[42] Diniz do Nascimento L, Moraes AAB, Costa KSD, Pereira Galúcio JM, Taube PS, Costa CML, Neves Cruz J, de Aguiar Andrade EH, Faria LJG, (2020). Bioactive natural compounds and antioxidant activity of essential oils from spice plants: new findings and potential applications. Biomolecules.

[43] Goula AM, Adamopoulos KG (2012). A method for pomegranate seed application in food industries: Seed oil encapsulation. Food Bioprod Process.

[44] Eratte D, Wang B, Dowling K, Barrow CJ, Adhikari BP (2014). Complex coacervation with whey protein isolate and gum arabic for the microencapsulation of omega-3 rich tuna oil. Food Funct.

[45] Noello C, Carvalho AGS, Silva VM, Hubinger MD (2016). Spray dried microparticles of chia oil using emulsion stabilized by whey protein concentrate and pectin by electrostatic deposition. Food Res Int.

[46] Roger B, Lagarce F, Garcion E, Benoit JP (2010). Biopharmaceutical parameters to consider in order altering the fate of nanocarriers after oral delivery. Nanomed.

[47] McClements DJ, Rao J (2011). Food-grade nanoemulsions: formulation, fabrication, properties, performance, biological fate, and potential toxicity. Crit Rev Food Sci Nutr.

[48] Mohammed ET, Safwat GM (2020). Grape seed proanthocyanidin extract mitigates titanium dioxide nanoparticle (TiO_2_-NPs)-induced hepatotoxicity through TLR-4/NF-κB signaling pathway. Biol Trace Elem Res.

[49] Thapa BR, Walia A (2007). Liver function tests and their interpretation. Ind J Pediatr.

[50] Abdel-Wahhab MA, El-Nekeety AA, Hathout AS, Salman AS, Abdel-Aziem SH, Sabry BA, Hassan NS, Abdel-Aziz MS, Aly SE, Jaswir I (2020). Bioactive compounds from *Aspergillus niger* extract enhance the antioxidant activity and prevent the genotoxicity in aflatoxin B_1_-treated rats. Toxicon.

[51] Ahamed M, AlSalhi MS, Siddigui MKJ (2010). Silver nanoparticle applications on human health. Clin Chim Acta.

[52] Fartkhooni FM, Noori A, Mohammadi A (2016). Effects of titanium dioxide nanoparticles toxicity on the kidney of male rats. Int J Life Sci.

[53] Duan Y, Liu J, Ma L, Li N, Liu H, Wang J, Zheng L, Liu C, Wang X, Zhao X, Yan J, Wang S, Wang H, Zhang X, Hong F (2010). Toxicological characteristics of nanoparticulate anatase titanium dioxide in mice. Biomater.

[54] Antoni R, Johnston KL, Collins AL, Robertson MD (2018). Intermittent v continuous energy restriction differential effects on postprandial glucose and lipid metabolism following matched weight loss in overweight/obese participants. Br J Nutr.

[55] Chen Z, Han S, Zheng P, Zhou D, Zhou S, Jia G (2020). Effect of oral exposure to titanium dioxide nanoparticles on lipid metabolism in Sprague-Dawley rats. Nanoscale.

[56] Reiner Ž (2017). Hyper-triglyceridaemia and risk of coronary artery disease. Nat Rev Cardiol.

[57] Foroozandeh P, Aziz AA (2015). Merging worlds of nanomaterials and biological environment factors governing protein corona formation on nanoparticles and its biological consequences. Nanoscale Res Lett.

[58] Rikans LE, Hornbrook KR (1997). Lipid peroxidation, antioxidant protection and aging. Biochim Biophys Acta Mol Basis Dis.

[59] Reeves JF, Davies SJ, Dodd NJ, Jha AN (2008). Hydroxyl radicals (OH) are associated with titanium dioxide (TiO_2_) nanoparticle-induced cytotoxicity and oxidative DNA damage in fish cells. Mutat Res.

[60] Shi H, Magaye R, Castranova V, Zhao J (2013). Titanium dioxide nanoparticles : a review of current toxicological data. Part Fibre Toxicol.

[61] Latchoumycandane C, Mathur P (2002). Induction of oxidative stress in the rat testis after short-term exposure to the organochlorine pesticide methoxychlor. Arch Toxicol.

[62] Sharma P, Singh R, Jan M (2014). Dose-dependent effect of deltamethrin in testis, liver, and kidney of Wistar rats. Toxicol Int.

[63] Huerta-García E, Pérez-Arizti JA, Márquez-Ramírez SG, Delgado-Buenrostro NL, Chirino YI, Iglesias GG, López-Marure R (2014). Titanium dioxide nanoparticles induce strong oxidative stress and mitochondrial damage in glial cells. Free Radic Biol Med.

[64] Kroemer G, Galluzzi L, Brenner C (2007). Mitochondrial membrane permeabilization in cell death. Physiol Rev.

[65] Jebali R, Ben Salah-Abbès J, Abbès S, Hassan AM, Abdel-Aziem SH, El-Nekeety AA, Oueslati R, Abdel-Wahhab MA (2018). *Lactobacillus plantarum* alleviate aflatoxins (B_1_ and M_1_) induced disturbances in the intestinal genes expression and DNA fragmentation in mice. Toxicon.

[66] Peng X, Chen K, Chen J, Fang J, Cui H, Zuo Z, Deng J, Chen Z, Geng Y, Lai W (2016). Aflatoxin B_1_ affects apoptosis and expression of Bax, Bcl-2, and caspase-3 in thymus and bursa of fabricius in broiler chickens. Environ Toxicol.

[67] Duan XX, Ou JS, Li Y, Su JJ, Ou C, Yang C, Yue HF, Ban KC (2005). Dynamic expression of apoptosis-related genes during development of laboratory hepatocellular carcinoma and its relation to apoptosis. World J Gastroenterol.

[68] Bhattacharya K, Davoren M, Boertz J, Schins RP, Hoffmann E, Dopp E (2009). Titanium dioxide nanoparticles induce oxidative stress and DNA-adduct formation but not DNA-breakage in human lung cells. Part Fibre Toxicol.

[69] Kansara K, Patel P, Shah D, Shukla RK, Singh S, Kumar A, Dhawan A (2015). TiO_2_ nanoparticles induce DNA double strand breaks and cell cycle arrest in human alveolar cells. Environ Mol Mutagen.

[70] Wani MR, Shadab G (2020) Titanium dioxide nanoparticle genotoxicity: a review of recent in vivo and in vitro studies. Toxicol Ind Health 36:514–530 10.1177/0748233720936835 32962563

[71] Jin C, Tang Y, Fan XY, Ye XT, Li XL, Tang K, Zhang YF, Li AG, Yang YJ (2013) In vivo evaluation of the interaction between titanium dioxide nanoparticle and rat liver DNA. Toxicol Ind Health 29:235–244 10.1177/0748233713479898 23443408

[72] Brandão F, Fernández-Bertólez N, Rosário F, Bessa MJ, Fraga S, Pásaro E, Teixeira JP, Laffon B, Valdiglesias V, Costa C (2020). Genotoxicity of TiO_2_ nanoparticles in four different human cell lines (A549, HEPG2, A172 and SH-SY5Y). Nanomater (Basel).

[73] Kazimirova A, Baranokova M, Staruchova M, Drlickova M, Volkovova K, Dusinska M (2019) Titanium dioxide nanoparticles tested for genotoxicity with the comet and micronucleus assays in vitro, ex vivo and in vivo. Mutat Res 843:57–65 10.1016/j.mrgentox.2019.05.001 31421740

[74] Tavares AM, Louro H, Antunes S, Quarré S, Simar S, De Temmerman PJ, Verleysen E, Mast J, Jensen KA, Norppa H, Nesslany F, Silva MJ (2014). Genotoxicity evaluation of nanosized titanium dioxide, synthetic amorphous silica and multi-walled carbon nanotubes in human lymphocytes. Toxicol in Vitro.

[75] Sycheva LP, Zhurkov VS, Iurchenko VV, Daugel-Dauge NO, Kovalenko MA, Krivtsova EK, Durnev AD (2011) Investigation of genotoxic and cytotoxic effects of micro and nanosized titanium dioxide in six organs of mice in vivo. Mutat Res Genet Toxicol Environ Mutagen 726(1):8–14 10.1016/j.mrgentox.2011.07.010 21871579

[76] Li H, Ding F, Xiao L, Shi R, Wang H, Han W, Huang Z (2017). Food-derived antioxidant polysaccharides and their pharmacological potential in neurodegenerative diseases. Nutrients.

[77] Fadoju O, Ogunsuyi O, Akanni O, Alabi O, Alimba C, Adaramoye O, Cambier S, Eswara S, Gutleb AC, Bakare A (2019). Evaluation of cytogenotoxicity and oxidative stress parameters in male Swiss mice co-exposed to titanium dioxide and zinc oxide nanoparticles. Environ Toxicol Pharmacol.

[78] Attia AM, Nasr HM (2009). Dimethoate-induced changes in biochemical parameters of experimental rat serum and its neutralization by black seed Nigella sativa L oil Slovak. J Anim Sci.

[79] Valentini X, Rugira P, Frau A, Tagliatti V, Conotte R, Laurent S, Colet JM, Nonclercq DD (2019) Hepatic and renal toxicity induced by TiO_2_ nanoparticles in rats: a morphological and metabonomic study. J Toxicol Mar 3: 2019:5767012. 10.1155/2019/5767012. 10.1155/2019/5767012 PMC6421043 30941172

[80] Pialoux V, Mounier R, Brown AD, Steinback CD, Rawling JM, Poulin MJ (2009). Relationship between oxidative stress and HIF-1α mRNA during sustained hypoxia in humans. Free Radic Biol Med.

[81] Olmedo DG, Tasat DR, Evelson P, Guglielmotti MB, Cabrini RL (2008). Biological response of tissues with macrophagic activity to titanium dioxide. J Biomed Mater Res Part A.

[82] Avetisyan A, Markosian A, Petrosyan M, Sahakyan N, Babayan A, Aloyan S, Trchounian A (2017). Chemical composition and some biological activities of the essential oils from basil Ocimum different cultivars. BMC Complement Altern Med.

[83] Nikolić B, Mitić-Ćulafić D, Vuković-Gačić B, Knežević-Vukčević J (2019) Plant mono-terpenes camphor, eucalyptol, thujone, and DNA repair. In: Patel V, Preedy V (eds). Handbook of Nutrition, Diet, and Epigenetics. Springer, Cham. 10.1007/978-3-319-55530-0_106.

[84] Rafael TM, Yolanda GR, Patricia RC, Alfredo SM, Joel LM, Alejandra OZ, Rafael SG (2018). Antioxidant activity of the essential oil and its major terpenes of Satureja macrostema (Moc. and Sessé ex Benth.). Briq Pharmacogn Mag.

[85] Ghosh T, Srivastava SK, Gaurav A, Kumar A, Kumar P, Yadav AS, Pathania R, Navani NK (2019) A combination of linalool, vitamin c, and copper synergistically triggers reactive oxygen species and DNA damage and inhibits *Salmonella enterica* subsp. enterica serovar typhi and *Vibrio fluvialis.* Appl Environ Microbiol 85(4): e02487–18. 10.1128/AEM.02487-18. 10.1128/AEM.02487-18 PMC6365828 30552187

[86] Huo M, Cui X, Xue J, Chi G, Gao R, Deng X, Guan S, Wei J, Soromou LW, Feng H, Wang D (2013). Anti-inflammatory effects of linalool in RAW 264.7 macrophages and lipopolysaccharide-induced lung injury model. J Surg Res.

[87] Deepa B, Venkatraman Anuradha C (2013). Effects of linalool on inflammation, matrix accumulation and podocyte loss in kidney of streptozotocin-induced diabetic rats. Toxicol Mech Methods.

[88] De Andrade CJ, Andrade LR, Silvana SM, Pastore G, Jauregi P (2017). A novel approach for the production and purification of mannosylerythritol lipids (MEL) by *Pseudozyma tsukubaensis* using cassava wastewater as substrate. Sep Purif Technol.

[89] Li J, Zhang X, Huang H (2014). Protective effect of linalool against lipopoly-saccharide/d-galactosamine-induced liver injury in mice. Int Immunopharmacol.

[90] Paula JF, Farago PV, Ribas JLC, Spinardi GMS, Döll MP, Artoni RF, Zawadzki SF (2007). In vivo evaluation of the mutagenic potential of estragole and eugenol chemotypes of Ocimum selloi Benth essential oil Lat. Am J Pharm..

[91] Silva-Alves KS, Ferreira-da-Silva FW, Peixoto-Neves D, Viana-Cardoso KV, Moreira-Júnior L, Oquendo MB, Oliveira-Abreu K, Albuquerque AA, Coelho-de-Souza AN, Leal-Cardoso JH (2013). Estragole blocks neuronal excitability by direct inhibition of Na+ channels. Braz J Med Biol Res.

[92] Silva-Comar FM, Wiirzler LA, Silva-Filho SE, Kummer R, Pedroso RB, Spironello RA, Silva EL, Bersani-Amado CA, Cuman RK (2014). Effect of estragole on leukocyte behavior and phagocytic activity of macrophages. Evid Based Complement Alternat Med.

[93] Martins FT, Doriguetto AC, de Souza TC, de Souza KR, Dos Santos MH, Moreira ME, Barbosa LC (2008). Composition and anti-inflammatory and antioxidant activities of the volatile oil from the fruit peel of Garcinia brasiliensis. Chem Biodivers.

[94] Queiroz JC, Antoniolli AR, Quintans-Júnior LJ, Brito RG, Barreto RS, Costa EV, da Silva TB, Prata AP, de Lucca WJr, Almeida JR , Lima JT, Quintans JS (2014). Evaluation of the anti-inflammatory and antinociceptive effects of the essential oil from leaves of Xylopia laevigata in experimental models. Sci World J.

[95] Xie Q, Li F, Fang L, Liu W, Gu C (2020) The antitumor efficacy of β-Elemene by changing tumor inflammatory environment and tumor microenvironment. Biomed Res Int. 10.1155/2020/6892961 10.1155/2020/6892961 PMC7054771 32149121

[96] Bai Z, Yao C, Zhu J, Xie Y, Ye XY, Bai R, Xie T (2021). Anti-tumor drug discovery based on natural product β-elemene anti-tumor mechanisms and structural modification. Molecules.

[97] Liu JS, Che XM, Chang S, Qiu GL, He SC, Fan L, Zhao W, Zhang ZL, Wang SF (2015). β-elemene enhances the radiosensitivity of gastric cancer cells by inhibiting Pak1 activation. World J Gastroenterol.

[98] Mao Y, Zhang J, Hou L, Cui X (2013). The effect of beta-elemene on alpha-tubulin polymerization in human hepatoma HepG2 cells. Chin J Cancer Res.

[99] K Zhou L Wang R Cheng X Liu S Mao 2017 Yan Y (2017) Elemene increases autophagic apoptosis and drug sensitivity in human cisplatin (DDP)-resistant lung cancer cell line SPC-A-1/DDP by inducing beclin-1 expression Oncol Res10.3727/096504017X14954936991990 10.3727/096504017X14954936991990 28550680

[100] Zhu T, Li X, Luo L, Wang X, Li Z, Xie P, Gao X, Song Z, Su J, Liang G (2015) Reversion of malignant phenotypes of human glioblastoma cells by β-elemene through β-catenin-mediated regulation of stemness-, differentiation- and epithelial-to-mesenchymal transition-related molecules. J Transl Med 13:356. 10.1186/s12967-015-0727-2 10.1186/s12967-015-0727-2 PMC4642639 26563263

[101] Li QQ, Lee RX, Liang H, Wang G, Li JM, Zhong Y, Reed E (2013). β-Elemene enhances susceptibility to cisplatin in resistant ovarian carcinoma cells via downregulation of ERCC-1 and XIAP and inactivation of JNK. Int J Oncol.

[102] Zhang J, Zhang HD, Chen L, Sun DW, Mao C, Chen W, Wu JZ, Zhong SL, Zhao JH, Tang JH (2014). β-elemene reverses chemoresistance of breast cancer via regulating MDR-related microRNA expression. Cell Physiol Biochem.

[103] Ebenyi LN, Ibiam UA, Aja PM (2012). Effects of *Allium sativum* extract on paracetamol induced hepatotoxicity in albino rats. IRJBB.

[104] El-Banna H, Soliman M, Al-Wabel N (2013). Hepatoprotective effects of thymus and salvia essential oils on paracetamol induced toxicity in rats. J Phys Pharm Adv.

[105] Edenharder R, Grȕnhage D (2003). Free radical scavenging abilities of flavonoids as mechanism of protection against mutagenicity induced by tert-butyl hydroperoxide or cumene hydroperoxide in *Salmonella typhimurium* TA102. Mutat Res.

[106] Paramasivan P, Kankia IH, Langdon SP, Deeni YY (2019) Emerging role of nuclear factor erythroid 2-related factor 2 in the mechanism of action and resistance to anticancer therapies. Cancer Drug Resist 2:490–515 10.20517/cdr.2019.57 PMC8992506 35582567

[107] De Flora S, Ferguson RL (2005) Overview of mechanisms of cancer chemo preventive agents. Mutat Res 591:8–15 10.1016/j.mrfmmm.2005.02.029 16107270

[108] Minj S, Anand S (2020) Whey proteins and its derivatives: bioactivity, functionality, and current applications. Dairy 1(3):233–258

[109] Gad AS, Khadrawy YA, El-Nekeety AA, Mohamed SR, Hassan NS,_,_ Abdel-Wahhab MA (2011) Antioxidant activity and hepatoprotective effects of whey protein and *spirulina* in rats. Nutr 27(5):582–589 10.1016/j.nut.2010.04.002 20708378

[110] Aboshanab MH, El-Nabarawi MA, Teaima MH, El-Nekeety AA, Abdel-Aziem SH, Hassan NS, Abdel-Wahhab MA (2020) Fabrication, characterization and biological evaluation of silymarin nanoparticles against carbon tetrachloride-induced oxidative stress and genotoxicity in rats. Int J Pharm 587:119639. 10.1016/j.ijpharm.2020.119639 10.1016/j.ijpharm.2020.119639 32673772

[111] Liu W, Wang G, Yakovlev AG (2002) Identification and functional analysis of the rat caspase-3 gene promoter. J Biol Chem 277(10):8273–8278 10.1074/jbc.M110768200 11773055

[112] Hassan MA, El-Nekeety AA, Abdel-Aziem SH, Hassan NS, Abdel-Wahhab MA (2019) Zinc citrate incorporation with whey protein nanoparticles alleviate the oxidative stress complication and modulate gene expression in the liver of rats. Food Chem Toxicol 125:439–45 10.1016/j.fct.2019.01.026 30711718

[113] Yonguc GN, Dodurga Y, Kurtulus A, Boz B, Acar K (2012) Caspase 1, Caspase 3, TNF-alpha, p53, and Hif1-alpha gene expression status of the brain tissues and hippocampal neuron loss in short-term dichlorvos exposed rats. Mol Biol Rep 39(12):10355–10360 10.1007/s11033-012-1913-4 23053939

